# Supplementary material for: Genome-Wide Identification of the Pectate Lyase Gene Family in Potato and Expression Analysis under Salt Stress
Source: Plants (Basel). 2024 May 11;13(10):1322. doi: 10.3390/plants13101322 (PMC11125077; doi:10.3390/plants13101322)
Supplement: Supplementary file 1 [file plants-13-01322-s001.zip › plants-2968369-supplementary.pdf]

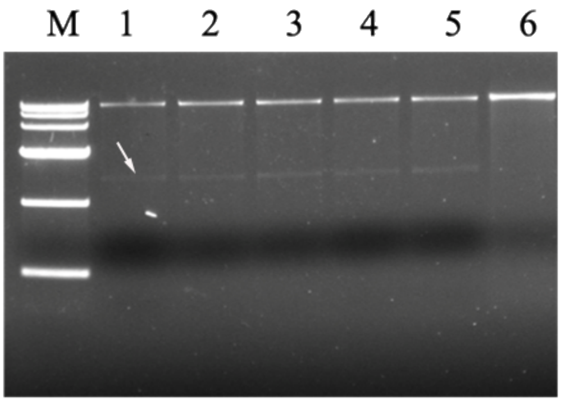

Figure S1: Enzyme digestion of the plant expression vector super1300-StPL18; M: DL15000; 1-5: Enzyme digestion of the recombinant plasmid super1300-StPL; 6. super1300-StPL18 plasmid.
